# Induction of sustained remission in early inflammatory arthritis with the combination of infliximab plus methotrexate: the DINORA trial

**DOI:** 10.1186/s13075-018-1667-z

**Published:** 2018-08-09

**Authors:** Tanja Alexandra Stamm, Klaus Peter Machold, Daniel Aletaha, Farideh Alasti, Peter Lipsky, David Pisetsky, Robert Landewe, Desiree van der Heijde, Alexandre Sepriano, Martin Aringer, Dimitri Boumpas, Gerd Burmester, Maurizio Cutolo, Wolfgang Ebner, Winfried Graninger, Tom Huizinga, Georg Schett, Hendrik Schulze-Koops, Paul-Peter Tak, Emilio Martin-Mola, Ferdinand Breedveld, Josef Smolen

**Affiliations:** 1 Section for Outcomes Research, Center for Medical Statistics, Informatics, and Intelligent Systems, Medical University of Vienna, Spitalgasse 23, 1090 Vienna, Austria; 2 Department of Medicine III, Division of Rheumatology, Medical University of Vienna, Waehringer Guertel 18-20, 1090 Vienna, Austria; 3 RILITE Research Institute, 250 W Main Street, Charlottesville, Virginia, 22902 USA; 4 Medical Research Service Durham VA Medical Center, and Duke University Medical Center, 151G Durham VA Medical Center, 508 Fulton Street, Durham, North Carolina 27705 USA; 5 Department of Medicine, Division of Rheumatology, Academic Medical Center Amsterdam, Amsterdam, The Netherlands; 6 Department of Rheumatology, Leiden University Medical Centre, Albinusdreef 2, PO Box 9600, 2300 RC Leiden, The Netherlands; 7 Division of Rheumatology, Department of Medicine III, University Medical Center and Faculty of Medicine Carl Gustav Carus at the TU Dresden, Fetscherstrasse 74, 01309 Dresden, Germany; 8 Rheumatology Medical School University of Crete, Heraklion and Joint Rheumatology Program, National and Kapodestrian University of Athens, Athens, Greece; 9 Department of Rheumatology and Clinical Immunology, Charité - University Medicine Berlin, Free University and Humboldt University Berlin, Berlin, Germany; 10 Research Laboratory and Division of Rheumatology, Department of Internal Medicine, University of Genova, Viale Benedetto XV, 6, 16132 Genoa, Italy; 11 Department of Internal Medicine, Centre for Rheumatic Diseases, Hietzing Hospital, Wolkersbergenstraße 1, 1130 Vienna, Austria; 12 Department of Rheumatology, Medical University of Graz, Auenbruggerplatz 15, 8036 Graz, Styria Austria; 13 Department of Internal Medicine 3, Rheumatology and Immunology, Friedrich-Alexander-University Erlangen-Nürnberg (FAU) and Universitätsklinikum Erlangen, Ulmenweg 18, 91054 Erlangen, Germany; 14 Division of Rheumatology and Clinical Immunology, Department of Internal Medicine IV, Ludwig Maximilians University of Munich, Pettenkoferstraße 8a, 80336 Munich, Germany; 15 Amsterdam Rheumatology and Immunology Center, Academic Medical Centre, University of Amsterdam, Amsterdam, the Netherlands; 16 Department of Medicine, Cambridge University, Cambridge, UK; 17 Department of Rheumatology, Ghent University, Ghent, Belgium; 18 GlaxoSmithKline Research & Development, Stevenage, UK; 19 Hospital Universitario La Paz, Paseo de la Castellana 261, 28046 Madrid, Spain; 20 Leiden University Medical Center, Albinusdreef 2, PO Box 9600, 2300 RC Leiden, The Netherlands

**Keywords:** Clinical remission, Early arthritis, Rheumatoid arthritis

## Abstract

**Background:**

In the present study, we explored the effects of immediate induction therapy with the anti-tumour necrosis factor (TNF)α antibody infliximab (IFX) plus methotrexate (MTX) compared with MTX alone and with placebo (PL) in patients with very early inflammatory arthritis.

**Methods:**

In an investigator-initiated, double-blind, randomised, placebo-controlled, multi-centre trial (ISRCTN21272423, http://www.isrctn.com/ISRCTN21272423), patients with synovitis of 12 weeks duration in at least two joints underwent 1 year of treatment with IFX in combination with MTX, MTX monotherapy, or PL randomised in a 2:2:1 ratio. The primary endpoint was clinical remission after 1 year (sustained for at least two consecutive visits 8 weeks apart) with remission defined as no swollen joints, 0–2 tender joints, and an acute-phase reactant within the normal range.

**Results:**

Ninety patients participated in the present study. At week 54 (primary endpoint), 32% of the patients in the IFX + MTX group achieved sustained remission compared with 14% on MTX alone and 0% on PL. This difference (*p* < 0.05 over all three groups) was statistically significant for IFX + MTX vs PL (*p* < 0.05), but not for IFX + MTX vs MTX (*p* = 0.10), nor for MTX vs PL (*p* = 0.31). Remission was maintained during the second year on no therapy in 75% of the IFX + MTX patients compared with 20% of the MTX-only patients.

**Conclusions:**

These results indicate that patients with early arthritis can benefit from induction therapy with anti-TNF plus MTX compared with MTX alone, suggesting that intensive treatment can alter the disease evolution.

**Trial registration:**

The trial was registered at http://www.isrctn.com/ISRCTN21272423 on 4 October 2007 (date applied)/12 December 2007 (date assigned). The first patient was included on 24 October 2007.

**Electronic supplementary material:**

The online version of this article (10.1186/s13075-018-1667-z) contains supplementary material, which is available to authorized users.

## Background

Rheumatoid arthritis (RA) is a severe chronic inflammatory joint disease that can lead to joint damage and functional impairment. Early therapy with disease-modifying anti-rheumatic drugs (DMARDs) can improve outcomes and limit joint damage and irreversible loss of physical function [1–3]. With the advent of newer therapeutic agents and treatment strategies [4, 5], the goal of remission is achievable in a proportion of patients [6–8]. Importantly, patients in clinical remission usually do not accrue additional joint damage [9, 10]. Despite these benefits of early therapy, drug-free remission is not attainable in the majority of patients [11, 12].

Early in the course of RA, a unique stage called the “window of opportunity” may exist. During this stage, key steps in pathogenesis may be reversible, with DMARD therapy blocking progression to full disease manifestations and potentially leading to sustained remission [13, 14]. Several findings provide support for the window of opportunity hypothesis: an increase in the risk of persistent disease after several months of arthritis symptoms [15, 16]; differences in immunological abnormalities in very early compared with established disease [17, 18]; and the ability of early treatment with a tumour necrosis factor (TNF) inhibitor plus methotrexate (MTX) to allow some patients with RA to achieve a drug-free remission [19, 20]. Information on the existence of the window of opportunity on the basis of current data is limited, however, since some studies did not have a double-blind design, evaluated only a very small number of patients and/or were performed only in a single centre. Furthermore, recent data obtained in patients with early disease suggest that, in those fulfilling the classification criteria of RA, drug-free remission after such induction therapy may be uncommon [12, 21].

Very little is known about the pathogenic processes operative in very early inflammatory arthritis, especially in those subjects who do not meet the classification criteria of RA [17, 18, 22]. Since remission due to MTX therapy alone is rare [23], we reasoned that MTX monotherapy might not be sufficient to induce lasting remission, even at this early stage of disease. Moreover, even though the presence of rheumatoid factor (RF) and anti-citrullinated protein antibodies (ACPA) has been found to identify subjects at increased risk of progressing to RA [24], we elected not to limit entry to subjects that had developed these biomarkers, but rather to examine a broader group of subjects who had developed unexplained inflammatory arthritis within the past 3 months in order to determine whether the presence of these antibodies or even the classification of RA altered the likelihood of progressing to RA despite intense therapy. The goal of this study, therefore, was to determine whether intense therapy with MTX plus infliximab (IFX) compared with MTX alone or placebo had the capacity to induce long-lasting drug-free remission in subjects with a very short period of inflammatory arthritis symptoms who had not received prior DMARD therapy.

## Methods

### Study design

The Definitive Intervention in New Onset Rheumatoid Arthritis (DINORA) study was a double-blind, randomised, placebo-controlled, multi-centre, investigator-initiated trial of the effects of anti-TNFα chimeric monoclonal antibody IFX in combination with MTX in patients with very early inflammatory arthritis and was conducted at 14 rheumatology centres across Europe (three in Austria, four in the Netherlands, four in Germany, and one each in Greece, Italy, and Spain). The study design is depicted in Additional file 1: Figure SA. The trial was registered at http://www.isrctn.com/ISRCTN21272423. Patient recruitment started in October 2007 and ended in February 2012. The study was conducted in accordance with the Declaration of Helsinki. Ethical committees of each institution approved the study and all patients gave written informed consent.

### Patients and randomisation

Patients were eligible for the trial if they had symptom duration of 2 to 12 weeks and had synovial swelling present in at least two joints (66 joint count); at least one joint must have been a metacarpophalangeal, proximal interphalangeal, or metatarsophalangeal (MTP) joint; MTP joints only were considered insufficient for inclusion. Baseline visits were scheduled if clinical joint swelling (arthritis) by history was present for 12 weeks and confirmed at two pre-treatment visits between week 2 and week 12 (Additional file 1: Figure SA). Patients with a positive purified protein derivative (PPD) test or chest radiograph performed at screening suggesting tuberculosis, malignancy, chronic infectious disease, elevated liver enzymes, or patients who were pregnant or planning to become pregnant within 6 months after the last infusion were excluded. Furthermore, patients with a distinct diagnosis made after a routine diagnostic work-up, such as a connective tissue disease, psoriatic arthritis, gout, pseudogout, reactive arthritis, or parvovirus arthritis, were not eligible. Thus, only patients with undifferentiated arthritis or early RA [25] were enrolled in the trial.

### Procedures of the study

Patients were randomised into three groups in a 2:2:1 ratio by a computer generated randomisation list to infliximab plus methotrexate (IFX + MTX), MTX monotherapy (MTX), or placebo (PL). For randomisation, patients were stratified for the use of glucocorticoids (users versus non-users, see below) and the presence of ACPA (> 7 units, measured by enzyme-linked immunosorbent assay (ELISA)) or high titre RF (> 50 IU/ml by nephelometry), determined in a central laboratory. Local investigators were blinded to the results of the central RF and ACPA testing and were also discouraged from having these tests performed on site. For reasons of blinding, a “double-dummy-like” administration of study medication was pursued. Every patient was treated with tablets containing MTX or PL and with infusions containing IFX or PL. The study medication code was kept blinded in patients who discontinued prematurely. Patients were followed until week 106. For rescue therapy for patients who discontinued treatment, the protocol recommended leflunomide (20 mg daily without a loading dose) or sulfasalazine (up to 3000 mg/day) with or without low-dose glucocorticoids.

Patients received treatment with IFX + MTX, MTX alone, or PL. In addition, supportive therapy appropriate at this early stage of arthritis was allowed in all three treatment groups. This therapy included non-steroidal anti-inflammatory drugs and, if necessary, glucocorticoids at a dose of no more than 10 mg/day prednisone or equivalent. MTX was dosed orally according to a rapid dose escalation scheme: treatment was started at 10 mg/week and increased to 25 mg/week in three steps with 2-week intervals except in cases of intolerance. IFX was administered by intravenous infusions at a dose of 3 mg/kg at 0, 2, and 6 weeks, and at 5 mg/kg every 8 weeks thereafter (and thus at higher than the minimal dose approved for maintenance therapy).

All core set variables were assessed at every visit. These variables included swollen and tender joint counts (SJC and TJC; using a 66- and 68-joint count, respectively), patient and evaluator global assessments (PGA and EGA, on a 100-mm visual analogue scale (VAS)), patient pain assessment (by VAS), erythrocyte sedimentation rate (ESR; mm/h), C-reactive protein (CRP; mg/dl), American College of Rheumatology (ACR) 20, 50, and 70% response rates [26], and the Health Assessment Questionnaire Disability Index (HAQ) [27]. Furthermore, composite measures of disease activity, such as Clinical and Simplified Disease Activity Index (CDAI and SDAI) [28] and Disease Activity Score 28 (DAS28) using 28-joint counts and ESR [29] were calculated.

Radiographs of hands and feet were taken at baseline, 6 months, 1 year, and 2 years and scored independently using the Sharp-van-der-Heijde (SvdH) method [30] by two readers who were blinded to patient characteristics and group allocation but who were aware of the chronological order of the films. The joint space narrowing (JSN) and erosion scores as well as their sum, representing the total score, were evaluated. The average score of the two readers was used for the analyses. In addition, random-effects models were fitted with and without imputation and by taking into account the scores from both readers and the interaction between treatment allocation and study visit to assess if the rate of radiological progression between the three treatment groups was significantly different.

### Endpoints

Persistent clinical remission at weeks 46 and 54 compared between all three treatment groups was taken as the primary endpoint. Clinical remission was defined as follows: at two consecutive visits, no swollen joint (66-joint count), 0 to at most 2 tender joints (68-joint count but counting unilateral MTPs as one joint), and a CRP level within the normal range (< 0.5 mg/dl) or a normal ESR (< 25 mm/h). At the time of the study design, the ACR/European League Against Rheumatism (EULAR) remission criteria [26] had not yet been developed. The criteria chosen here, however, are consistent with these criteria; similar to the Boolean or index-based remission criteria, they do not allow for more than two affected joints (sum of swollen or tender) and require a normal CRP [10, 28].

In all patients, the last infusion of IFX was planned at week 54 (or earlier, as specified below), whereas MTX was continued at the same dose until week 58 and then tapered in all patients over 4 weeks (weekly reduction by 5 mg/week, last dose at week 62). IFX + MTX, MTX, or PL was discontinued earlier if clinical remission was attained at two consecutive visits after the 14-week visit. Thus, for patients who reached clinical remission at two or more consecutive visits before week 54 (sustained remission), IFX (or PL) was stopped and MTX (or PL) tapered beginning after the second visit in remission (first planned possible IFX withdrawal at week 30; Additional file 1: Figure SA). Since the patients would not know on which regimen they had achieved remission, no blinded infusions were continued from that time-point onward. However, as mentioned above, to qualify for the primary endpoint, patients had to have sustained remission until week 54 irrespective of early withdrawal. The study was continued until week 106 without further study medication to evaluate long-term maintenance of remission; blinding of initial treatment assignment remained intact.

### Statistical analysis

The sample size calculation is described in Additional file 2: Supplement S1. Descriptive statistics were used for baseline characteristics and demographic data. We applied a strategy of step-wise hierarchical hypothesis testing [31] to control for type I error of the primary and key secondary (SDAI and DAS28 scores) endpoints. The primary endpoint was analysed at the fixed 46- to 54-week time point (because two visits were needed to define sustained/persistent remission) using Fisher’s exact test. Persistent clinical remission at weeks 46 and 54 was evaluated as a categorical variable (in remission or not) and Fisher’s exact test was calculated for differences over all three treatment groups. For the primary endpoint analysis, we applied non-responder imputation (NRI) for dichotomous variables from those visits onward at which patients had missing data or for patients who started rescue DMARD therapy, and the last observation carried forward (LOCF) method for continuous variables. In case of a significant result regarding the overall difference between all three groups, the primary endpoint was subsequently assessed comparing each of two groups, respectively: group 1 (IFX + MTX) versus 3 (PL), 1 (IFX + MTX) versus 2 (MTX), and 2 (MTX) versus 3 (PL). Longitudinal data analysis of clinical remission is described in Additional file 2: Supplement S2. Secondary endpoints were tested at years 1 and 2 using either Fisher’s exact test for categorical variables or Kruskal-Wallis test for continuous data.

## Results

### Demographic data and patient flow

**Fig. 1:**
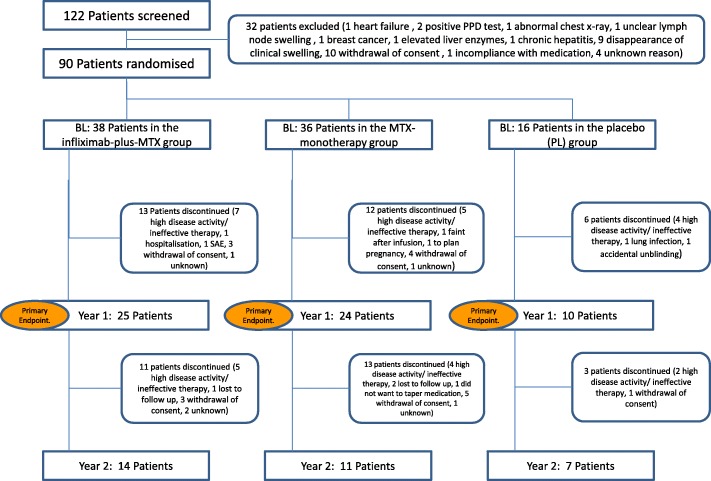
Patient flow chart; the patient flow of the DINORA study. BL baseline, MTX methotrexate, PPD purified protein derivative, SAE serious adverse event

Of the 122 screened patients, 90 were randomised and dosed at the baseline visit (Fig. 1). Baseline characteristics and demographic data are described in Table 1. Table 2 depicts the number of patients in clinical remission in the three treatment groups at 6 months, 1 year, and 2 years. Early withdrawal within the first 3 months was seen in three patients in the IFX + MTX group , in two in the MTX group, and in one patient in the PL group.

**Table 1:** Baseline characteristics of the study sample

	IFX + MTX	MTX	PL	*P* value
Number of patients (*n*)	38	36	16	0.4440
Female	26 (68.4%)	28 (77.8%)	9 (56.3%)	0.2833
Age (years), mean ± SD	52.1 ± 14.1	52.9 ± 14.0	54.4 ± 11.2	0.9170
Symptom duration (weeks)^a^, mean ± SD	10.3 ± 2.3	9.4 ± 2.3	9.8 ± 1.8	0.0722
Rheumatoid factor positive	13 (34.2%)	13 (36.1%)	7 (43.8%)	0.7988
Patients who used steroids prior to the study	24 (63.2%)	22 (61.1%)	9 (56.3%)	0.8931
Anti-citrullinated protein antibody positive	18 (47.4%)	16 (44.4%)	7 (43.8%)	0.9563
Patients who meet the ACR/EULAR 2010 classification criteria for RA, 2010 [25]	26 (68%)	19 (53%)	12 (75%)	0.2135
Patients who meet the 1987 ARA classification criteria for RA [35]	22 (58%)	19 (53%)	9 (56%)	0.9049
Health Assessment Questionnaire (0–3)	0.9 ± 0.7	0.9 ± 0.6	0.7 ± 0.7	0.2903
Disease Activity Score 28 (DAS28; based on ESR)	5.0 ± 1.4	4.8 ± 1.3	4.7 ± 1.1	0.8464
Simplified Disease Activity Index (SDAI)	34.3 ± 23.8	31.1 ± 14.4	27.5 ± 20.0	0.4771
Clinical Disease Activity Index (CDAI)	25.1 ± 14.7	26.2 ± 13.9	23.5 ± 11.9	0.8951
Swollen joint count (0–28)	7.2 ± 5.7	6.50 ± 5.1	7.4 ± 4.6	0.7048
Tender joint count (0–28)	9.2 ± 7.3	10.3 ± 7.2	7.8 ± 5.6	0.5263
Visual analogue scale pain (mm)	44.0 ± 29.3	44.2 ± 24.3	44.6 ± 22.7	0.9595
Patient global assessment (mm)	48.6 ± 29.0	47.8 ± 24.7	39.6 ± 21.0	0.5274
Evaluator/physician global assessment (mm)	38.6 ± 18.3	46.3 ± 22.3	44.6 ± 20.7	0.3627
C-reactive protein (mg/dl)	1.71 ± 2.40	1.18 ± 1.88	0.98 ± 1.28	0.5567
Erythrocyte sedimentation rate (ESR; mm/h)	23.2 ± 20.3	20.3 ± 21.2	20.4 ± 12.6	0.8129
Total Sharp-van-der-Heide score	2.8 ± 5.4	3 ± 3.8	4.6 ± 8.6	0.4816
Erosion score	1.2 ± 1.8	1.6 ± 2.2	2.2 ± 4.2	0.6019
Joint space narrowing score	1.6 ± 3.8	1.4 ± 2.2	2.4 ± 4.4	0.5658

Data are shown as mean ± standard deviation or *n* (%) as appropriate

The parameters showed no significant differences between the three groups at baseline

Tables with additional data on baseline characteristics as well as 1-year data for the patients who were in remission at 1 year are provided in Additional file 2 (Tables SC and SD)

*ACR* American College of Rheumatology, *ARA* American Rheumatism Association, *EULAR* European League Against Rheumatism, *IFX* infliximab, *MTX* methotrexate, *PL* placebo, *RA* rheumatoid arthritis

^a^Symptom duration refers to the first visit when the patients presented themselves at the centres. At the baseline visit, symptom duration of all patients was 12 weeks because baseline visits were scheduled at this time to ensure persistent arthritis for 12 weeks. Patients who had no residual arthritis at the baseline visit were excluded

**Table 2:** Clinical characteristics of the study sample at 6 months, 1 year, and 2 years

	IFX + MTX *n* = 38	MTX *n* = 36	PL *n* = 16
Clinical remission (primary endpoint), no. of patients in remission (%)			
6 months	10 (26%)	6 (17%)	0
1 year	12 (32%)	5 (14%)	0
2 years	9 (24%)	1 (3%)	3 (19%)
Other definitions of remission, no. of patients in remission (%)			
Disease Activity Score 28 (DAS28)			
6 months	20 (53%)	11 (31%)	1 (6%)
1 year	24 (63%)	13 (36%)	3 (19%)
2 years	23 (61%)	11 (31%)	5 (31%)
Simplified Disease Activity Index (SDAI)			
6 months	16 (42%)	9 (25%)	1 (6%)
1 year	18 (47%)	13 (36%)	1 (6%)
2 years	18 (47%)	13 (36%)	4 (25%)
ACR/EULAR Boolean			
6 months	15 (40%)	8 (22%)	0
1 year	13 (34%)	9 (25%)	1 (6%)
2 years	13 (34%)	10 (28%)	4 (25%)
ACR improvement, responders			
ACR20			
6 months	20 (53%)	18 (50%)	4 (25%)
1 year	22 (58%)	22 (61%)	3 (19%)
2 years	19 (50%)	19 (53%)	3 (19%)
ACR50			
6 months	16 (42%)	13 (36%)	1 (6%)
1 year	17 (45%)	16 (44%)	3 (19%)
2 years	14 (37%)	15 (42%)	3 (19%)
ACR70			
6 months	15 (40%)	6 (17%)	1 (6%)
1 year	14 (37%)	11 (31%)	2 (13%)
2 years	13 (34%)	11 (31%)	3 (19%)
Other secondary outcome parameters (mean ± SD)			
Pain			
6 months	17.3 ± 20.3	22.5 ± 25.2	42.7 ± 31.0
1 year	20.9 ± 23.8	18.3 ± 25.3	45.7 ± 31.8
2 years	23.0 ± 25.1	23.3 ± 29.8	43.5 ± 32.8
Swollen joints (28 joints)			
6 months	2.3 ± 5.2	2.1 ± 4.5	4.9 ± 5.6
1 year	2.3 ± 5.2	2.1 ± 4.3	5.0 ± 5.6
2 years	2.8 ± 5.6	2.4 ± 4.5	5.1 ± 5.6
Tender joints (28 joints)			
6 months	2.9 ± 5.9	4.9 ± 6.2	7.0 ± 6.4
1 year	2.5 ± 5.6	4.2 ± 6.0	7.2 ± 6.8
2 years	3.4 ± 6.5	4.0 ± 6.1	7.1 ± 7.0
Patient global visual analogue scale (VAS; mm)			
6 months	17.7 ± 6.5	23.1 ± 24.6	35.1 ± 28.2
1 year	21.2 ± 24.0	18.4 ± 24.7	38.0 ± 29.3
2 years	24.3 ± 25.3	24.8 ± 30.0	35.6 ± 29.5
Evaluator global VAS (mm)			
6 months	16.1 ± 22.0	17.2 ± 24.1	34.6 ± 28.0
1 year	14.1 ± 20.8	17.7 ± 24.6	39.3 ± 29.8
2 years	16.6 ± 24.1	18.4 ± 24.7	34.5 ± 31.3
C-reactive protein (mg/dl)			
6 months	0.5 ± 0.9	0.6 ± 1.0	0.8 ± 0.9
1 year	0.5 ± 0.9	0.5 ± 1.0	0.7 ± 0.8
2 years	0.6 ± 1.1	0.6 ± 1.0	0.5 ± 0.8
Erythrocyte sedimentation rate (mm)			
6 months	14.6 ± 12.2	17.8 ± 12.5	14.9 ± 6.9
1 year	14.6 ± 12.3	18.7 ± 13.0	18.3 ± 9.7
2 years	16.5 ± 14.1	17.6 ± 11.2	16.5 ± 10.7
Health Assessment Questionnaire (HAQ)			
6 months	0.30 ± 0.45	0.57 ± 0.64	0.54 ± 0.67
1 year	0.33 ± 0.46	0.52 ± 0.62	0.61 ± 0.66
2 years	0.41 ± 0.52	0.58 ± 0.61	0.62 ± 0.65
X-rays^a^			
6 months	−0.02 ± 0.88	0.07 ± 0.23	0.41 ± 1.53
1 year	0.18 ± 1.06	0.16 ± 0.44	0.0 ± 0.41
2 years	0.36 ± 0.95	0.28 ± 0.67	0.63 ± 1.31

Missing data for continuous variables were imputed using last observation carried forward (LOCF). LOCF was also applied from the time points onwards when patients received other DMARDs as rescue therapy. The denominator for the percentages given is the number of patients initially included in each group and stays consistent for each year

*ACR* American College of Rheumatology, *EULAR* European League Against Rheumatism, *IFX* infliximab, *MTX* methotrexate, *PL* placebo

^a^Mean change of scores ± SD from baseline of Sharp-van-der-Heijde (SvdH) for patients with complete follow-up data at each time point

### Clinical remission at 1 year

**Fig. 2:**
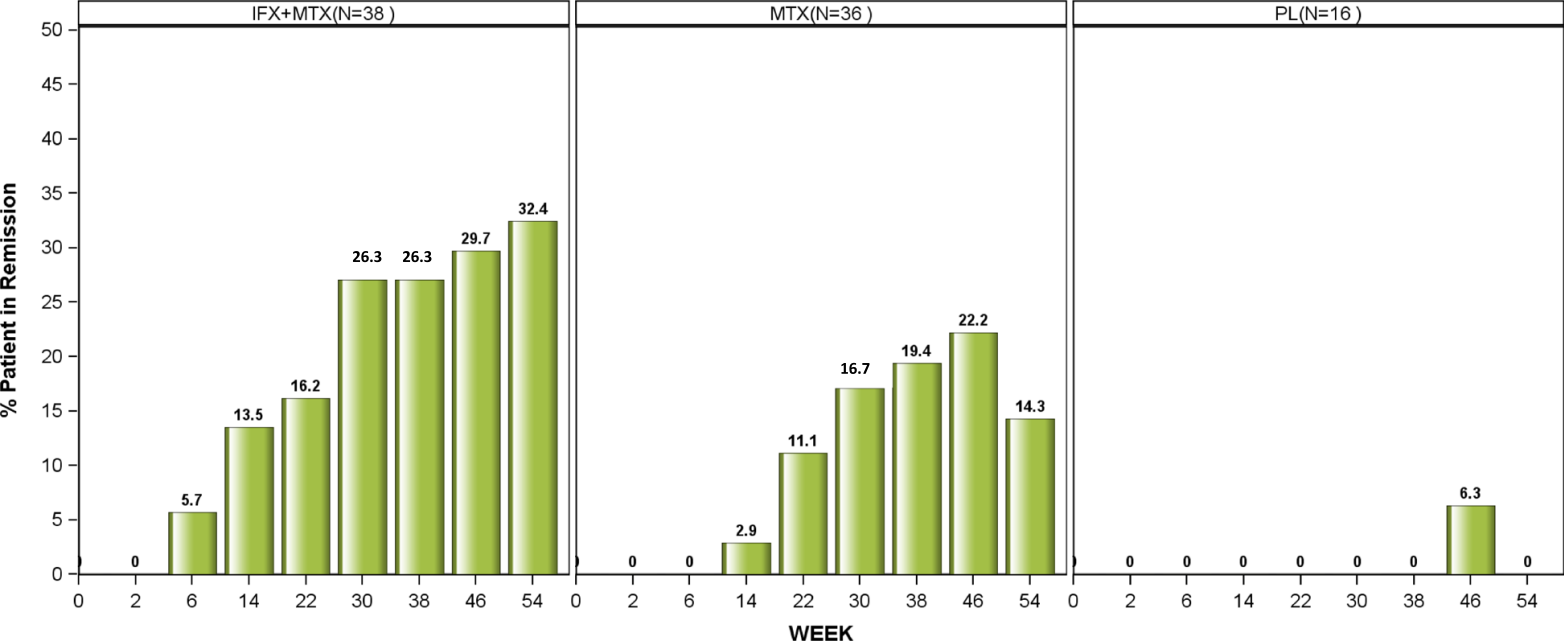
Proportions of remissions; percentage of patients who achieved remission for each time point across the treatment groups. The denominator stays constant, meaning for example in the infliximab (IFX) + methotrexate (MTX) group that the proportion of patients in remission is always divided by 38 (the total number of patients included in this group at baseline). PL placebo

At week 54 (primary endpoint), more patients in the IFX + MTX group (12/38, 32%) achieved sustained clinical remission compared with 5/36 (14%) on MTX alone and none (0/16, 0%) on PL. The overall difference across all three treatment groups showed statistical significance (*p* < 0.05; Additional file 1: Figure SB). Upon subsequent pairwise comparisons, differences in rates of sustained clinical remission were significant between IFX + MTX and PL (treatment effect: 32%; *p* < 0.05), but not between the IFX + MTX and MTX (treatment effect 18%; *p* > 0.05), nor between MTX and PL (treatment effect 14%; *p* > 0.05). Figure 2 shows sustained remission rates as defined for the primary outcome in a cumulative way over time for each of the treatment groups. By week 30, 10 patients (26%) treated with IFX + MTX had already achieved clinical remission at two consecutive visits and all these patients sustained clinical remission until weeks 46 and 54. It is noteworthy that almost one in three patients receiving IFX + MTX, but only one in seven in the MTX group and none on PL had achieved sustained clinical remission at 1 year. The number needed to treat (NNT) to achieve one additional sustained remission at 52 weeks with IFX + MTX was 3 compared with placebo, while the NNT for MTX alone versus placebo was 7; NNT was 6 when comparing IFX + MTX with MTX alone. The results of the longitudinal data analysis are described in Additional file 2: Supplement S3.

### Clinical remission at the end of year 2

Maintenance of a remission state until the end of year 2 differed significantly across the three groups (*p* = 0.0210); only 20% of patients in remission on MTX monotherapy at 54 weeks maintained remission, whereas this was the case in 75% of those attaining this state on IFX + MTX despite withdrawal of therapy (*p* = 0.0140 for the comparison between IFX + MTX and MTX groups; Table 2). The NNT to achieve sustained remission at 2 years with IFX + MTX was 2 compared with treatment with MTX alone, and 2 compared with placebo; the NNT for MTX alone versus placebo was 5.

### Changes in disease activity and core set variables

At week 54, the proportions of patients with DAS28 < 2.6 and ACR20 responses were significantly different over all three groups (*p* < 0.01 for DAS28 < 2.6 and *p* < 0.05 for ACR20), as well as pain scores measured on a VAS (*p* < 0.05). The stratified differences between treatment groups revealed significance between the IFX + MTX and MTX groups (*p* < 0.05 for DAS28 < 2.6), IFX + MTX and PL (*p* < 0.001 for DAS28 < 2.6; *p* < 0.05 for ACR20; *p* < 0.05 for pain scores), and between MTX and PL (*p* < 0.01 for ACR20; *p* < 0.01 for pain scores).

### Patients classified as RA or non-RA at baseline

In the IFX + MTX group, 8/26 (30.8%) of the patients classified as RA [25] achieved clinical remission at the primary endpoint (1 year) compared with 4/12 (33.3%) of the patients who did not fulfil RA classification criteria. In the MTX group, 2/19 (10.5%) of the patients classified as RA reached the primary endpoint at 1 year compared with 3/17 (17.6%) of the patients who did not fulfil RA criteria (data not shown). When looking at the 2-year outcome in the IFX + MTX group, 5/26 (19%) of the patients classified as RA [25] achieved clinical remission compared with 4/12 (33.3%) of the patients who did not fulfil RA classification criteria. In the MTX group, 1/19 (5%) patient classified as RA reached the remission at 2 years compared with none who did not fulfil RA criteria (data not shown). Two of the three patients who were in remission after 2 years in the PL group fulfilled the RA classification criteria at baseline, and all three PL patients were ACPA and RF negative. Thus, there was no difference in outcomes whether patients fulfilled the ACR/EULAR classification criteria [25] or not. The presence of RF made a significant difference in the remission frequency at the 2-year time only point (Chi square test; *p* = 0.0399); the presence of ACPA made no significant difference in the frequency of remission at 6 months, 1 year, or 2 years.

### Radiographic changes

Mean change from baseline of the Sharp-van-der-Heide scores [30] did not reveal any noteworthy differences between the three treatment groups (Table 2 and Additional file 2: Figure SC and Table SA).

### Adverse events

The occurrences of adverse events (AEs) and serious adverse events (SAEs) are depicted in Table 3. There were no statistically significant differences in the number of patients with AEs between the three treatment groups (Fisher’s exact test; data not shown).

**Table 3:** Patients with adverse events (AEs) and serious adverse events (SAEs)

	Total *n* = 90	IFX + MTX *n* = 38	MTX *n* = 36	PL *n* = 16
Adverse events, *n* (%)				
Infectious/parasitic disease	31 (35%)	19 (50%)	9 (25%)	3 (19%)
Malignancy	1 (1%)	0	1 (3%)	0
Disease of blood, blood-forming organs, and immune mechanisms (except arthritis)	1 (1%)	1 (3%)	0	0
Endocrine, nutritional, and metabolic diseases	9 (10%)	2 (5%)	5 (14%)	2 (12%)
Disease of the nervous system	17 (19%)	6 (16%)	9 (25%)	2 (13%)
Diseases of the eye	3 (3%)	1 (3%)	2 (6%)	0
Diseases of circulatory system	16 (18%)	6 (16%)	7 (19%)	3 (19%)
Diseases of respiratory system	43 (48%)	23 (61%)	16 (44%)	4 (25%)
Diseases of the digestive system	37 (41%)	15 (39%)	17 (47%)	5 (31%)
Diseases of the skin and subcutaneous tissue	25 (28%)	12 (32%)	8 (22%)	5 (31%)
Diseases of musculoskeletal system and connective tissue	30 (33%)	15 (39%)	9 (25%)	6 (38%)
Diseases of urogenital system (pregnancy, childbirth, and puerperium)	4 (4%)	2 (5%)	2 (6%)	0
Symptoms, signs, and abnormal clinical and laboratory findings not elsewhere classified	27 (30%)	11 (29%)	11 (31%)	5 (31%)
Injury, poisoning and certain other consequences of external causes	9 (10%)	6 (16%)	3 (8%)	0
External causes of morbidity	1 (1%)	1 (3%)	0	0
Total		120	99	35
SAEs (*n* = 12)				
Hospitalisation due to different reasons^a^		4	1	3
Fainted during blood collection prior to administration of study drug		1	0	0
Significantly raised transaminase levels		1	0	0
Hematuria, followed by a diagnosis of bladder cancer		0	1	0
Hypertensive episode 1 h after the last infusion with study drug		0	1	0

Only 4 (0.9%) of all reported AEs and no SAEs were considered definitely related to the study drug. 155 AEs (37%) and no SAEs were regarded as possibly/probably related to one of the study drugs. No participant died during the 2-year study period. Two SAEs were related to infections (1 gastrointestinal, 1 genitourinary); however, in both cases patients were on PL only. One of these SAEs was related to a malignancy (bladder cancer on MTX monotherapy) and none to tuberculosis

*IFX* infliximab, *MTX* methotrexate, *PL* placebo

^a^Hyperglycemia (PL), diarrhoea (PL), urinary tract infection (IFX + MTX), urinary tract infection with fever (PL), MTX pneumonitis (opportunistic) infection (IFX + MTX), significant flare of disease activity (IFX + MTX), myocardial infarction more than half a year after last study drug (MTX), and biliary pancreatitis in a time after the study medication (IFX + MTX)

## Discussion

The present trial in patients with very early arthritis yields several important findings on the effects of treatment of early inflammatory arthritis with DMARD therapy. First, as in the SAVE trial [32], our study indicated that spontaneous remission (on placebo and supportive treatment alone) occurred only very rarely in patients with undifferentiated arthritis or very early RA of 12 weeks duration. Secondly, we observed that therapy with anti-TNF plus MTX, while significantly different from PL regarding all outcomes, produced more than twice as many stringently defined remissions when compared with MTX alone (32% and 14%, respectively); while the trend was clear, the difference in response rates did not achieve statistical significance (*p* = 0.10). Thirdly, we found that the majority of those patients who had remission on IFX + MTX therapy maintained remission even after withdrawal of all therapies (overall 24% still had clinical remission at 2 years); in contrast, 80% of those on MTX alone lost their remission state, leaving only 3% of MTX-treated patients in remission at 2 years. Fourthly, the vast majority of patients who achieved sustained drug-free remission had already attained this state within 30 weeks, indicating that the necessity for longer treatment durations to achieve remission does not increase drug-free remission rates. Together, these findings suggest that once joint inflammation is clinically manifest, symptomatic therapy does not impact on the course of disease, that initiation of DMARD therapy is warranted to improve outcomes, and that early intensive treatment with anti-TNF + MTX leads to drug-free remission in 1 of 4 patients. When we assessed other remission definitions, such as SDAI or Boolean remission criteria or DAS28 < 2.6, we saw even higher remission rates in the IFX + MTX group at 1 year (34–63%), but this was also the case for the MTX group and the difference across all three groups was not significant, except for DAS28 < 2.6. These findings might be due to the heterogeneity of the patients in our study; furthermore, our data suggest that aggressive therapy is not always necessary in very early inflammatory arthritis for obtaining sustained drug-free remission, although we did not find any respective predictive markers (data not shown).

In addition to delineating the effects of DMARD therapy on early inflammatory arthritis, this study provides important new information on the validity of the window of opportunity hypothesis. This hypothesis proposes that a short period of intensive therapy early in the disease course may reverse the disease process and produce long-term benefits. Indeed, in this study, almost one-third of the patients receiving IFX + MTX achieved sustained remission, with 9/12 (75%) of those who attained remission in the first year maintaining this state 1 year later without any treatment (or a total of 9/38 (24%) of the randomised patients). This outcome contrasts with that of patients treated with MTX alone since only 1 in 7 achieved remission in the first year, with the majority subsequently losing this state; as a result, only 3% of patients treated with MTX alone had a sustained remission at 2 years. Thus, anti-TNF + MTX induction treatment demonstrated a clear advantage compared with supportive therapy at 1 year and compared with MTX-only therapy at 2 years; in contrast, induction with MTX alone failed to show a significantly better response than placebo. Importantly, the active therapies, in particular anti-TNF plus MTX, did not appear to cause major serious adverse events in this early arthritis patient population, with the two observed serious adverse events occurring in patients on supportive care.

While one-third of patients treated with IFX + MTX had a favourable outcome, two-thirds of the patients treated with these agents did not attain remission within the first year. This result is disappointing and could argue against the window of opportunity hypothesis. It is important to note, however, that our results pertain only to the combination of IFX + MTX. As shown in other studies, RA patients can differ in their response to biological agents, perhaps based on their mode of action, and we do not know which patients will respond best to a given targeted therapy [33]. On the other hand, the study outcome is quite promising, since 1 of 4 patients with early arthritis did reach drug-free remission after a short course of an induction therapy with these agents. Importantly, in contrast to other randomised controlled trials in early, although established, RA, we did not observe a gradual decline in responders over the second year [21, 34], suggesting a true abrogation or reversal of the disease process.

Our study has several limitations. First, the study may have been underpowered to show a significant difference between the IFX + MTX and MTX-alone groups. For the purposes of this study, we estimated placebo and spontaneous remission rates on the basis of previous observational studies, and the lack of precise data on remission rates from these trials could have led to difficulties in balancing MTX alone and anti-TNF + MTX responses. Although our results indicate that the majority of patients failed to achieve sustained remission on anti-TNF + MTX, the difference between anti-TNF + MTX and MTX alone in the first year might have reached statistical significance with a larger sample size. Of note, in this regard, patients treated with MTX alone did not maintain remission until the end of year 2 while IFX + MTX patients did, suggesting relevant differences in the effects of combination therapy compared with monotherapy. The results of our study need to be interpreted with caution; however, they do suggest that early intensive treatment may alter the course of inflammatory arthritis. One notable unexpected finding was the difficulty of recruiting patients into this study which contrasted with our experience from a previous study [32]. We attempted to enrol very early arthritis patients who had to consent to their participation in a long-term (2 years) study at one of their first visits to the rheumatology centre before many of them had the opportunity to recognise and accept the implications of a diagnosis of inflammatory arthritis and possibly RA. Our experiences may be useful in planning future investigator-driven studies with larger sample sizes in this very early arthritis population of patients.

## Conclusions

In conclusion, our study provides encouraging evidence that a short-term induction therapy with a TNF inhibitor plus MTX can yield long-term benefit in a considerable proportion of patients with early arthritis, even after cessation of all therapy. In contrast, the data presented indicate that MTX alone will not produce responses that are maintained over time. Placebo or supportive treatment alone neither improves nor reverses disease; these findings represent further evidence that spontaneous remission is rare once the signs and symptoms of RA have emerged. While the current study involves only TNF as a target of biological therapy, the data nevertheless strongly support the possibility that patients with early inflammatory arthritis may have a window of opportunity in which disease reversal is possible.

## Additional files

Additional file 1: Supplemental figures SA and SB. (PPTX 101 kb)

Additional file 2: Supplemental files. (DOCX 47 kb)

## Abbreviations

ACPA: Anti-citrullinated protein antibodies
ACR: American College of Rheumatology
CRP: C-reactive protein
DAS28: Disease Activity Score 28
DINORA: Definitive Intervention in New Onset Rheumatoid Arthritis
DMARD: Disease-modifying anti-rheumatic drug
EGA: Evaluator global assessment
ESR: Erythrocyte sedimentation rate
EULAR: European League Against Rheumatism
HAQ: Health Assessment Questionnaire Disability Index
IFX: Infliximab
MTP: metatarsophalangeal
MTX: Methotrexate
NNT: Number needed to treat
PGA: Patient global assessment
RA: Rheumatoid arthritis
RF: Rheumatoid factor
SDAI: Simplified Disease Activity Index
SJC: Swollen joint count
TJC: Tender joint count
TNF: Tumour necrosis factor
VAS: Visual analogue scale

## References

[1] van der Heide A, Jacobs JW, Bijlsma JW, Heurkens AH, van Booma-Frankfort C, van der Veen MJ, Haanen HC, Hofman DM, van Albada-Kuipers GA, ter Borg EJ (1996). The effectiveness of early treatment with “second-line” antirheumatic drugs. A randomized, controlled trial. Ann Intern Med.

[2] Nell VK, Machold KP, Eberl G, Stamm TA, Uffmann M, Smolen JS. Benefit of very early referral and very early therapy with disease-modifying anti-rheumatic drugs in patients with early rheumatoid arthritis. Rheumatology (Oxford). 2004; Epub (ahead of print) 10.1093/rheumatology/keh199 15113999

[3] Lard LR, Visser H, Speyer I, vander Horst-Bruinsma IE, Zwinderman AH, Breedveld FC, Hazes J (2001). Early versus delayed treatment in patients with recent-onset rheumatoid arthritis: comparison of two cohorts who received different treatment strategies. AmJMed.

[4] Grigor C, Capell H, Stirling A, McMahon AD, Lock P, Vallance R, Kincaid W, Porter D (2004). Effect of a treatment strategy of tight control for rheumatoid arthritis (the TICORA study): a single-blind randomised controlled trial. Lancet.

[5] Goekoop-Ruiterman YP, de Vries-Bouwstra JK, Kerstens PJ, Nielen MM, Vos K, van Schaardenburg D, Speyer I, Seys PE, Breedveld FC, Allaart CF, et al. DAS-driven therapy versus routine care in patients with recent-onset active rheumatoid arthritis. Ann Rheum Dis. 2010;69(1):65–9. 10.1136/ard.2008.097683 19155234

[6] Singh JA, Furst DE, Bharat A, Curtis JR, Kavanaugh AF, Kremer JM, Moreland LW, O'Dell J, Winthrop KL, Beukelman T (2012). 2012 update of the 2008 American College of Rheumatology recommendations for the use of disease-modifying antirheumatic drugs and biologic agents in the treatment of rheumatoid arthritis. Arthritis Care Res.

[7] Smolen JS, Landewe R, Breedveld FC, Buch M, Burmester G, Dougados M, Emery P, Gaujoux-Viala C, Gossec L, Nam J (2014). EULAR recommendations for the management of rheumatoid arthritis with synthetic and biological disease-modifying antirheumatic drugs: 2013 update. Ann Rheum Dis.

[8] Mierau M, Schoels M, Gonda G, Fuchs J, Aletaha D, Smolen JS (2007). Assessing remission in clinical practice. Rheumatology(Oxford).

[9] Felson DT, Smolen JS, Wells G, Zhang B, van Tuyl LH, Funovits J, Aletaha D, Allaart CF, Bathon J, Bombardieri S (2011). American College of Rheumatology/European league against rheumatism provisional definition of remission in rheumatoid arthritis for clinical trials. Ann Rheum Dis.

[10] Aletaha D, Funovits J, Breedveld FC, Sharp J, Segurado O, Smolen JS (2009). Rheumatoid arthritis joint progression in sustained remission is determined by disease activity levels preceding the period of radiographic assessment. Arthritis Rheum.

[11] Tanaka Y, Takeuchi T, Mimori T, Saito K, Nawata M, Kameda H, Nojima T, Miyasaka N, Koike T, investigators RRRs (2010). Discontinuation of infliximab after attaining low disease activity in patients with rheumatoid arthritis: RRR (remission induction by Remicade in RA) study. Ann Rheum Dis.

[12] Emery P, Hammoudeh M, FitzGerald O, Combe B, Martin-Mola E, Buch MH, Krogulec M, Williams T, Gaylord S, Pedersen R (2014). Sustained remission with etanercept tapering in early rheumatoid arthritis. N Engl J Med.

[13] Furst DE (2004). Window of opportunity. J Rheumatol.

[14] Raza K, Saber TP, Kvien TK, Tak PP, Gerlag DM (2012). Timing the therapeutic window of opportunity in early rheumatoid arthritis: proposal for definitions of disease duration in clinical trials. Ann Rheum Dis.

[15] Green M, Marzo-Ortega H, McGonagle D, Wakefield R, Proudman S, Conaghan P, Gooi J, Emery P (1999). Persistence of mild, early inflammatory arthritis: the importance of disease duration, rheumatoid factor, and the shared epitope. Arthritis Rheum.

[16] van der Linden MP, le Cessie S, Raza K, van der Woude D, Knevel R, Huizinga TW, van der Helm-van Mil AH (2010). Long-term impact of delay in assessment of patients with early arthritis. Arthritis Rheum.

[17] Raza K, Falciani F, Curnow SJ, Ross EJ, Lee CY, Akbar AN, Lord JM, Gordon C, Buckley CD, Salmon M (2005). Early rheumatoid arthritis is characterized by a distinct and transient synovial fluid cytokine profile of T cell and stromal cell origin. Arthritis Res Ther.

[18] de Hair MJ, van de Sande MG, Ramwadhdoebe TH, Hansson M, Landewe R, van der Leij C, Maas M, Serre G, van Schaardenburg D, Klareskog L (2014). Features of the synovium of individuals at risk of developing rheumatoid arthritis: implications for understanding preclinical rheumatoid arthritis. Arthritis Rheumatol.

[19] Goekoop-Ruiterman YP, de Vries-Bouwstra JK, Allaart CF, van Zeben D, Kerstens PJ, Hazes JM, Zwinderman AH, Peeters AJ, de Jonge-Bok JM, Mallee C (2007). Comparison of treatment strategies in early rheumatoid arthritis: a randomized trial. Ann Intern Med.

[20] Quinn MA, Conaghan PG, O'Connor PJ, Karim Z, Greenstein A, Brown A, Brown C, Fraser A, Jarret S, Emery P (2005). Very early treatment with infliximab in addition to methotrexate in early, poor-prognosis rheumatoid arthritis reduces magnetic resonance imaging evidence of synovitis and damage, with sustained benefit after infliximab withdrawal: results from a twelve-month randomized, double-blind, placebo-controlled trial. Arthritis Rheum.

[21] Emery P, Burmester GR, Bykerk VP, Combe BG, Furst DE, Barre E, Karyekar CS, Wong DA, Huizinga TW (2015). Evaluating drug-free remission with abatacept in early rheumatoid arthritis: results from the phase 3b, multicentre, randomised, active-controlled AVERT study of 24 months, with a 12-month, double-blind treatment period. Ann Rheum Dis.

[22] Choi IY, Karpus ON, Turner JD, Hardie D, Marshall JL, de Hair MJ, Maijer KI, Tak PP, Raza K, Hamann J (2017). Stromal cell markers are differentially expressed in the synovial tissue of patients with early arthritis. PLoS One.

[23] Goekoop-Ruiterman YP, Vries-Bouwstra JK, Allaart CF, van Zeben D, Kerstens PJ, Hazes JM, Zwinderman AH, Ronday HK, Han KH, Westedt ML (2005). Clinical and radiographic outcomes of four different treatment strategies in patients with early rheumatoid arthritis (the BeSt study): a randomized, controlled trial. Arthritis Rheum.

[24] Visser H, le Cessie S, Vos K, Breedveld FC, Hazes JM (2002). How to diagnose rheumatoid arthritis early: a prediction model for persistent (erosive) arthritis. Arthritis Rheum.

[25] Aletaha D, Neogi T, Silman AJ, Funovits J, Felson DT, Bingham CO, Birnbaum NS, Burmester GR, Bykerk VP, Cohen MD (2010). 2010 rheumatoid arthritis classification criteria: an American College of Rheumatology/European League Against Rheumatism collaborative initiative. AnnRheumDis.

[26] Felson DT, Anderson JJ, Boers M, Bombardier C, Furst D, Goldsmith C (1995). American College of Rheumatology preliminary definition of improvement in rheumatoid arthritis. Arthritis Rheum.

[27] Fries JF, Spitz P, Kraines RG, Holman HR (1980). Measurement of patient outcome in arthritis. Arthritis Rheum.

[28] Aletaha D, Ward MM, Machold KP, Nell VP, Stamm T, Smolen JS (2005). Remission and active disease in rheumatoid arthritis: defining criteria for disease activity states. Arthritis Rheum.

[29] Prevoo ML, 't Hof MA, Kuper HH, van Leeuwen MA, van de Putte LB, van Riel PL (1995). Modified disease activity scores that include twenty-eight-joint counts. Development and validation in a prospective longitudinal study of patients with rheumatoid arthritis. Arthritis Rheum.

[30] van der Heijde D (2000). How to read radiographs according to the sharp/van der Heijde method. J Rheumatol.

[31] Genovese MC, Kremer J, Zamani O, Ludivico C, Krogulec M, Xie L, Beattie SD, Koch AE, Cardillo TE, Rooney TP (2016). Baricitinib in patients with refractory rheumatoid arthritis. N Engl J Med.

[32] Machold KP, Landewe R, Smolen JS, Stamm TA, van der Heijde DM, Verpoort KN, Brickmann K, Vazquez-Mellado J, Karateev DE, Breedveld FC (2010). The stop arthritis very early (SAVE) trial, an international multicentre, randomised, double-blind, placebo-controlled trial on glucocorticoids in very early arthritis. AnnRheumDis.

[33] Smolen JS, Aletaha D (2015). Rheumatoid arthritis therapy reappraisal: strategies, opportunities and challenges. Nat Rev Rheumatol.

[34] Emery P, Breedveld FC, Hall S, Durez P, Chang DJ, Robertson D, Singh A, Pedersen RD, Koenig AS, Freundlich B (2008). Comparison of methotrexate monotherapy with a combination of methotrexate and etanercept in active, early, moderate to severe rheumatoid arthritis (COMET): a randomised, double-blind, parallel treatment trial. Lancet.

[35] Arnett FC, Edworthy SM, Bloch DA, McShane DJ, Fries JF, Cooper NS, Healey LA, Kaplan SR, Liang MH, Luthra HS (1988). The American Rheumatism Association 1987 revised criteria for the classification of rheumatoid arthritis. Arthritis Rheum.

