# Supplementary material for: Induction of sustained remission in early inflammatory arthritis with the combination of infliximab plus methotrexate: the DINORA trial
Source: Arthritis Res Ther. 2018 Aug 9;20:174. doi: 10.1186/s13075-018-1667-z (PMC6085639; doi:10.1186/s13075-018-1667-z)
Supplement: Supplementary file 1 — Supplemental figures SA and SB. (PPTX 101 kb) [file 13075_2018_1667_MOESM1_ESM.pptx]

## Slide 1
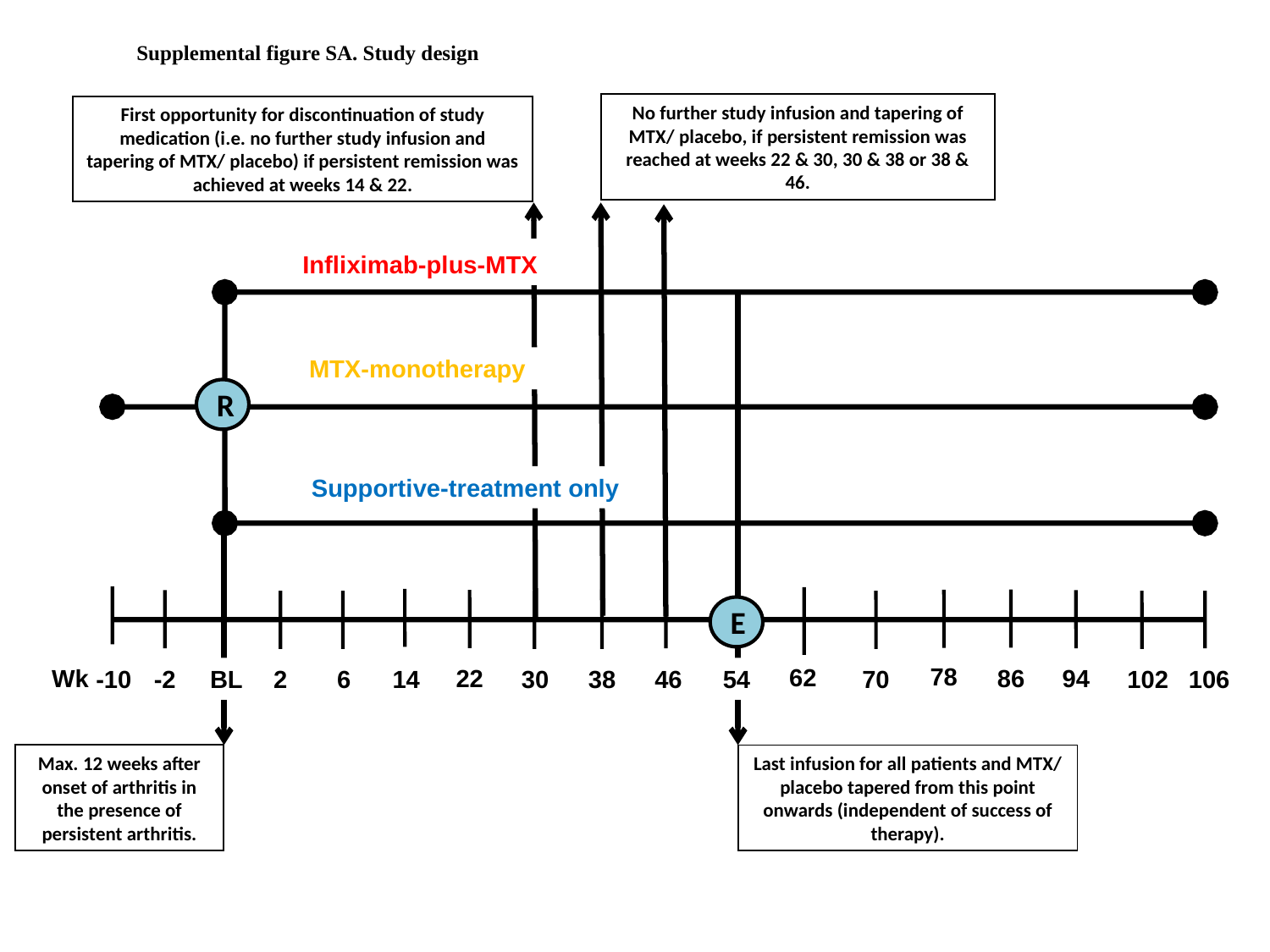

Supplemental figure SA. Study design
No further study infusion and tapering of MTX/ placebo, if persistent remission was reached at weeks 22 & 30, 30 & 38 or 38 & 46.
First opportunity for discontinuation of study medication (i.e. no further study infusion and tapering of MTX/ placebo) if persistent remission was achieved at weeks 14 & 22.
Infliximab-plus-MTX
MTX-monotherapy
R
Supportive-treatment only
E
78
62
22
86
Wk
94
-10
-2
BL
2
6
14
30
38
46
54
70
102
106
Max. 12 weeks after onset of arthritis in the presence of persistent arthritis.
Last infusion for all patients and MTX/ placebo tapered from this point onwards (independent of success of therapy).

## Slide 2
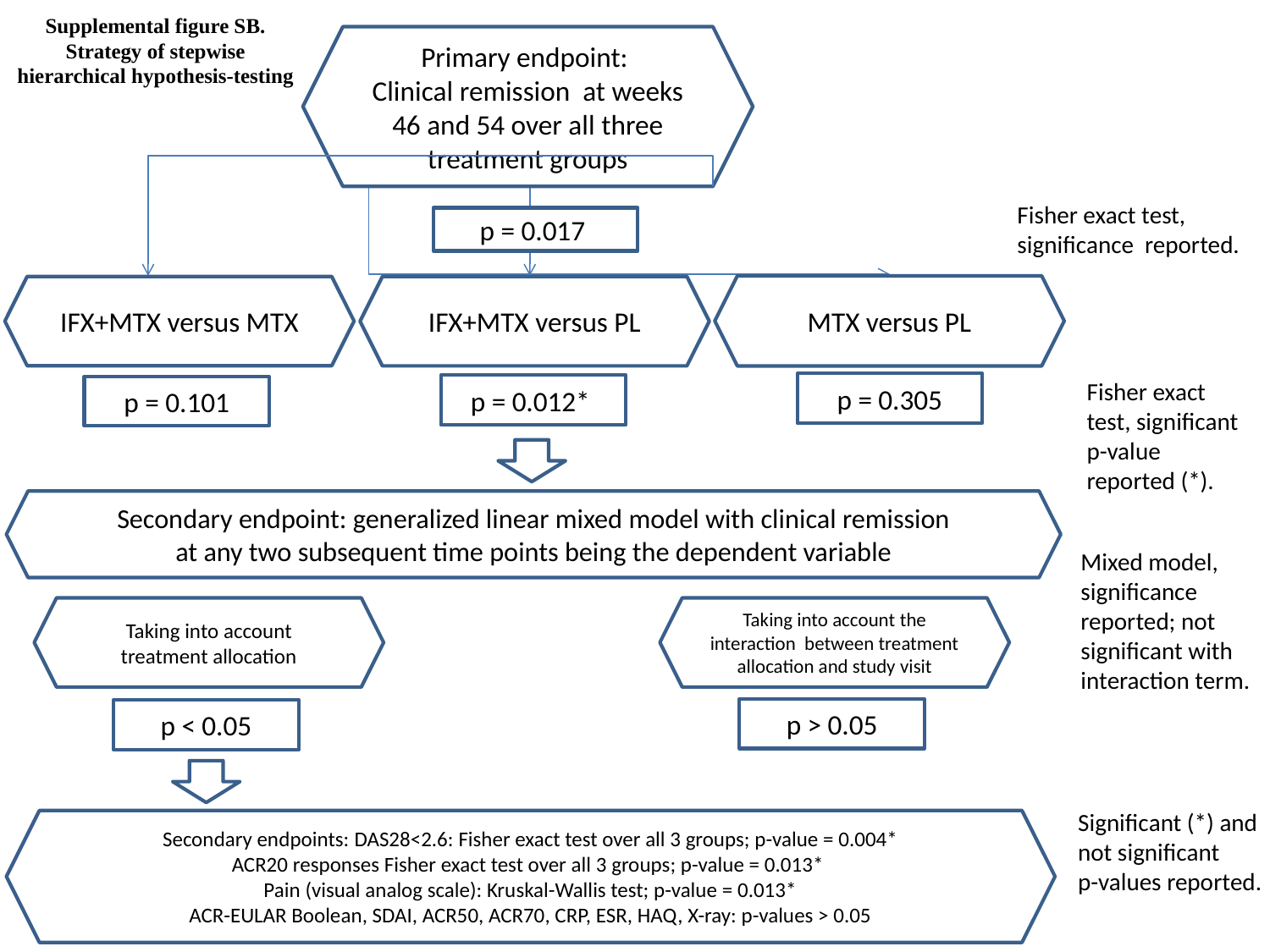

Supplemental figure SB. Strategy of stepwise hierarchical hypothesis-testing
Primary endpoint:
Clinical remission at weeks 46 and 54 over all three treatment groups
Fisher exact test, significance reported.
p = 0.017
MTX versus PL
IFX+MTX versus MTX
IFX+MTX versus PL
Fisher exact
test, significant
p-value
reported (*).
p = 0.305
p = 0.012*
p = 0.101
Secondary endpoint: generalized linear mixed model with clinical remission at any two subsequent time points being the dependent variable
Mixed model,
significance
reported; not
significant with
interaction term.
Taking into account treatment allocation
Taking into account the interaction between treatment allocation and study visit
p > 0.05
p < 0.05
Significant (*) and
not significant
p-values reported.
Secondary endpoints: DAS28<2.6: Fisher exact test over all 3 groups; p-value = 0.004*
ACR20 responses Fisher exact test over all 3 groups; p-value = 0.013*
Pain (visual analog scale): Kruskal-Wallis test; p-value = 0.013*
ACR-EULAR Boolean, SDAI, ACR50, ACR70, CRP, ESR, HAQ, X-ray: p-values > 0.05
