# Supplementary material for: Induction of sustained remission in early inflammatory arthritis with the combination of infliximab plus methotrexate: the DINORA trial
Source: Arthritis Res Ther. 2018 Aug 9;20:174. doi: 10.1186/s13075-018-1667-z (PMC6085639; doi:10.1186/s13075-018-1667-z)
Supplement: Supplementary file 2 — Supplemental files. (DOCX 47 kb) [file 13075_2018_1667_MOESM2_ESM.docx]

**Supplement/Appendix – Revised version R1**

**Supplement S1. Sample size calculation**

In the initial sample size calculation, we estimated that group sizes of 73 in the IFX+MTX and the MTX groups, respectively, were needed to achieve 80% power to detect a difference of 0.25 between the null hypothesis that both the MTX+IFX and MTX proportions are 0.55 and the alternative hypothesis that the proportion in MTX+IFX is 0.80 using a two-sided Chi-square test with continuity correction and a significance level of 0.025 (0.05/2). Furthermore, a group size of 12 in the PL was needed to achieve 80% power to detect a difference of 0.50 between the null hypothesis that both the PL and the IFX-MTX group proportions are 0.30 and the alternative hypothesis that the proportion in IFX+MTX is 0.80. However, since the time of the original study design, new studies were published which allowed re-calculating the sample size as described in the following paragraph.

These studies included the Stop Arthritis Very Early (SAVE) and the 3-week course of intramuscular glucocorticoid injections in patients with very early inflammatory polyarthritis (STIVEA) trials (1, 2). Therefore, we estimated 50% response rate in the IFX+MTX, 20% in the MTX and 10% in the PL groups, with the placebo response rate (spontaneous stringent remission) based on 8% of patients as seen in SAVE (1) and various early RA trials for MTX and anti-TNF plus MTX (3-6). With 80% power and a significance level of 0.05 to differentiate anti-TNF plus MTX from both MTX alone and PL, the sample size calculation resulted into a total of 90 patients (2:2:1 randomization) for the three treatment groups.

**Supplement S2. Description of the longitudinal data analysis of clinical remission**

In addition, longitudinal generalized linear mixed models with random intercepts were fitted, with clinical remission at two consecutive time points until weeks 46 and 54 being the dependent variable (sustained/persistent remission over time). The model parameters were estimated taking into account the treatment allocation and the interaction between treatment allocation and study visit. Generalized linear mixed models were chosen because they take into account between-individual heterogeneity, the longitudinal nature of the data and the within-subject correlations of the variables (7). In contrast to ordinary regression methods, generalized linear mixed models do not assume independent observations. Furthermore, numbers needed to treat (NNT) being defined as the inverse of the average number of patients who need to be treated for one additional benefit (clinical remission) compared between IFX+MTX, MTX alone and/or PL, were calculated.

**Supplement S3. Results of the longitudinal analysis of clinical remission**

Supplemental table B depicts the results of the generalized linear mixed model that revealed a statistically significant effect of IFX+MTX and MTX over one year (the time of the primary endpoint) for a patient to achieve clinical remission at two subsequent visits when compared to PL (p<0.05). The estimated odds ratios in supplemental table B indicate that a patient in the IFX+MTX group had a 35-times higher change to reach clinical remission compared to PL. Two further generalized linear mixed models, one model taking into account the interaction between treatment allocation and study visit and another model based on the MTX+IFX and the MTX groups only without the placebo group, did not reveal statistically significant contributions/effects of either IFX+MTX or MTX to achieve clinical remission (data not shown).

**Figure SA. Study design of the DINORA trial (included in the additional file 1)**

**Figure SB. Stepwise hierarchical hypothesis testing:** the strategy of stepwise hierarchical hypothesis testing used in the present study **(included in the additional file 1).**

**Figure SC. Total SvdH scores over time by treatment group:** descriptive statistics (graphical display) of mean (± standard deviation) of the absolute value of the total SvdH score (average between reader 1 and reader 2 without imputation) during 24 months of follow-up per treatment group. All three treatment groups had similar progression scores. Longitudinal analyses showed no significant effect of treatment with either IFN+MTX or MTX-only as compared to placebo in the rate of progression of the SvdH score over the 2-year follow-up as well as no significant difference in the rate of radiographic progression between treatment groups over the 2-years follow-up (interaction treatment-time p-value>0.15) (data not shown). The overall progression rate was relatively low even in the PL group.

******

**Table SA.** **Mean (±SD) of SvdH scores over time by treatment group without imputation:** the average between reader 1 and reader 2 was used.

| **Treatment arm** | **Baseline** | **6 months** | **12 months** | **24 months** |
| --- | --- | --- | --- | --- |
| IFN+MTX | 3.5 (6.7)  n=35 | 4.1 (7.2)  n=28 | 5.1 (8.2)  n=22 | 7.6 (10.8)  n=12 |
| MTX | 3.5 (4.3)  n=30 | 3.3 (3.7)  n=23 | 3.5 (4.0)  n=19 | 3.4 (3.5)  n=9 |
| Placebo | 3.8 (5.9)  n=14 | 4.5 (7.0)  n=11 | 5.4 (7.9)  n=7 | 9.3 (10.7)  n=4 |

**Table SB. Results of the generalized linear mixed model with clinical remission** **at two time points including week 54 being the dependent variable.** The model shows that patients in the IFX+MTX and MTX groups have a significantly higher change to reach clinical remission after one year compared to placebo (PL) which is the reference value.

The dependent variable y_i_ in the model is the likelihood of reaching clinical remission as defined in the study protocol (at two consecutive visits). *i* (=1, ...,*n*) indexed the patients. *j* (=1, ...,*ni*) indexed the study visit for patient *i*; x is the treatment allocation: y_i_ = random intercept_i_ + βx_ij_ + error_i_

A random intercept and a fixed slope were used. The model below was selected based on the lowest Akaike information criterion (AIC) which is a measure of the relative quality of statistical models.

| **Treatment allocation** | **Coefficient** | **95% CI (Coefficient)** | **Odds ratio** | **95% CI**  **(Odds ratio)** | **p-value** |
| --- | --- | --- | --- | --- | --- |
| IFX+MTX | 3.6 | 1.2, 5.9 | 35.0 | 3.3, 365.0 | 0.003 |
| MTX | 2.8 | 0.6, 5.0 | 16.5 | 1.8, 148.4 | 0.019 |
| PL (reference) | - | - | - | - | - |

**Table SC. Baseline characteristics of the patients who were in remission at year one.** Data are shown as mean (±SD) or absolute (relative) frequencies where appropriate. The number of patients classified as RA or non-RA at baseline who were in remission at year one are shown in a separate paragraph in the results section of the main body of the paper.

|  | **IFX+MTX** | **MTX** |
| --- | --- | --- |
| Number of patients in remission (N) | 12 | 5 |
| Female (%) | 4 (33.3) | 1 (20) |
| Age (years) | 52.8 (13.1) | 43.9 (11.9) |
| Symptom duration (weeks)* | 10.2 (1.7) | 10.8 (1.3) |
| Rheumatoid factor (% positive) | 3 (25) | 1 (20) |
| Anti-citrullinated protein antibodies (% positive) | 5 (41.7) | 2 (40) |
| Health Assessment Questionnaire (0-3) | 0.8 (0.7) | 0.6 (0.4) |
| Disease activity score 28 (DAS28; based on ESR) | 4.5 (1.4) | 3.4 (2.1) |
| Simplified disease activity index (SDAI) | 24.6 (5) | 19.6 (12.4) |
| Clinical disease activity index (CDAI) | 21.9 (13.7) | 17.6 (9.1) |
| Swollen joint count (0-28) | 7 (4.8) | 4.6 (3.1) |
| Tender joint count (0-28) | 8.2 (7.1) | 5.8 (6.7) |
| Visual analogue scale pain (mm) | 35.4 (29.5) | 34 (22) |
| Patient global assessment (mm) | 29.3 (26.5) | 39 (20.1) |
| Evaluator/Physician global assessment (mm) | 28.3 (16.4) | 33.4 (14.5) |
| C-reactive protein (CRP; mg/dl) | 1.6 (2.5) | 1.1 (0.8) |
| Erythrocyte sedimentation rate (ESR; mm/h) | 15 (15.7) | 5.7 (3.5) |
| Total Sharp-van-der-Heide score | 4.8 (7.8) | 0.8 (1.8) |
| Erosion score | 1.4 (1.4) | 0.4 (0.9) |
| Joint space narrowing score | 3.4 (7.3) | 0.4 (0.9) |

**Table SD. One year data of patients who were in remission at year one.** Data are shown as mean (±SD) or absolute (relative) frequencies where appropriate.

|  | **IFX+MTX** | **MTX** |
| --- | --- | --- |
| Number of patients (N) | 12 | 5 |
| Health Assessment Questionnaire (0-3) | 0.1 (0.2) | 0 (0.1) |
| Disease activity score 28 (DAS28; based on ESR) | 1.6 (0.5) | 1.1 (0.6) |
| Simplified disease activity index (SDAI) | 1.1 (0.8) | 1.1 (1.5) |
| Clinical disease activity index (CDAI) | 0.9 (0.8) | 0.9 (1.5) |
| Swollen joint count (0-28) | 0 | 0 |
| Tender joint count (0-28) | 0.2 (0.4) | 0.2 (0.5) |
| Visual analogue scale pain (mm) | 4.7 (6.8) | 1.8 (2.9) |
| Patient global assessment (mm) | 5.8 (7.1) | 4.3 (6.1) |
| Evaluator/Physician global assessment (mm) | 12.1 (2.6) | 2.3 (4.5) |
| C-reactive protein (CRP; mg/dl) | 0.2 (0.3) | 0.2 (0.4) |
| Erythrocyte sedimentation rate (ESR; mm/h) | 9 (6) | 6.8 (7) |
| Total Sharp-van-der-Heide score | 4.7 (7.5) | 0 (0) |
| Erosion score | 1.2 (1.2) | 0 (0) |
| Joint space narrowing score | 3.5 (7.0) | 0 (0) |

1. Machold KP, Landewe R, Smolen JS, Stamm TA, van der Heijde DM, Verpoort KN, et al. The Stop Arthritis Very Early (SAVE) trial, an international multicentre, randomised, double-blind, placebo-controlled trial on glucocorticoids in very early arthritis. AnnRheumDis. 2010;69(3):495-502.

2. Verstappen SM, McCoy MJ, Roberts C, Dale NE, Hassell AB, Symmons DP, et al. Beneficial effects of a 3-week course of intramuscular glucocorticoid injections in patients with very early inflammatory polyarthritis: results of the STIVEA trial. Ann Rheum Dis. 2010;69(3):503-9.

3. Smolen JS, Emery P, Fleischmann R, van Vollenhoven RF, Pavelka K, Durez P, et al. Adjustment of therapy in rheumatoid arthritis on the basis of achievement of stable low disease activity with adalimumab plus methotrexate or methotrexate alone: the randomised controlled OPTIMA trial. Lancet. 2014;383(9914):321-32.

4. Breedveld FC, Weisman MH, Kavanaugh AF, Cohen SB, Pavelka K, van Vollenhoven R, et al. The PREMIER study: A multicenter, randomized, double-blind clinical trial of combination therapy with adalimumab plus methotrexate versus methotrexate alone or adalimumab alone in patients with early, aggressive rheumatoid arthritis who had not had previous methotrexate treatment. Arthritis Rheum. 2006;54(1):26-37.

5. Keystone EC, Kavanaugh AF, Sharp JT, Tannenbaum H, Hua Y, Teoh LS, et al. Radiographic, clinical, and functional outcomes of treatment with adalimumab (a human anti-tumor necrosis factor monoclonal antibody) in patients with active rheumatoid arthritis receiving concomitant methotrexate therapy: a randomized, placebo-controlled, 52-week trial. Arthritis Rheum. 2004;50(5):1400-11.

6. Weinblatt ME, Keystone EC, Furst DE, Moreland LW, Weisman MH, Birbara CA, et al. Adalimumab, a fully human anti-tumor necrosis factor alpha monoclonal antibody, for the treatment of rheumatoid arthritis in patients taking concomitant methotrexate: the ARMADA trial. Arthritis Rheum. 2003;48(1):35-45.

7. Carriere I, Bouyer J. Choosing marginal or random-effects models for longitudinal binary responses: application to self-reported disability among older persons. BMC medical research methodology. 2002;2:15.
